# Advanced NSCLC Patients With EGFR T790M Harboring TP53 R273C or KRAS G12V Cannot Benefit From Osimertinib Based on a Clinical Multicentre Study by Tissue and Liquid Biopsy

**DOI:** 10.3389/fonc.2021.621992

**Published:** 2021-02-24

**Authors:** Yulong Fu, Anqi Wang, Jieqi Zhou, Wei Feng, Minhua Shi, Xiao Xu, Hongqing Zhao, Liming Cai, Jian Feng, Xuedong Lv, Xiaodong Zhang, Wenjing Xu, Zhengrong Zhang, Guoer Ma, Jian Wang, Tong Zhou, Dahai Zhao, Haohui Fang, Zeyi Liu, Jian-an Huang

**Affiliations:** ^1^ Department of Pulmonary and Critical Care Medicine, The First Affiliated Hospital of Soochow University, Suzhou, China; ^2^ Suzhou Key Laboratory for Respiratory Diseases, Suzhou, China; ^3^ Department of Respiratory Medicine, The Second Affiliated Hospital of Soochow University, Suzhou, China; ^4^ Department of Respiratory Medicine, Affiliated Suzhou Hospital of Nanjing Medical University, Suzhou, China; ^5^ Department of Respirology, Nanjing Medical University Affiliated Wuxi Second Hospital, Wuxi, China; ^6^ Department of Respiratory Medicine, Affiliated Hospital of Jiangnan University, Wuxi, China; ^7^ Department of Respiratory Medicine, Affiliated Hospital of Nantong University, Nantong, China; ^8^ Department of Respiratory Medicine, The Second Affiliated Hospital of Nantong University, Nantong, China; ^9^ Department of Medical Oncology, Nantong Tumor Hospital, Nantong, China; ^10^ Departments of Respiratory Medicine, Northern Jiangsu People’s Hospital, Clinical Medical College of Yangzhou University, Yangzhou, China; ^11^ Department of Respiratory Medicine, First People’s Hospital of Yangzhou City, Yangzhou, China; ^12^ Department of Respiratory Medicine, Affiliated Hospital of Jiangsu University, Zhenjiang, China; ^13^ Department of Respiratory Medicine, Zhenjiang First People’s Hospital, Zhenjiang, China; ^14^ Department of Oncology, Changzhou Cancer Hospital Affiliated to Soochow University, Changzhou, China; ^15^ Department of Respiratory and Critical Care Medicine, The Second Hospital of Anhui Medical University, Hefei, China; ^16^ Department of Respiratory Medicine, Anhui Chest Hospital, Hefei, China; ^17^ Institute of Respiratory Diseases, Soochow University, Suzhou, China

**Keywords:** non-small cell lung cancer, liquid biopsy, gene sequencing, epidermal growth factor receptor-tyrosine kinase inhibitor (EGFR-TKI), drug resistance

## Abstract

**Background:**

Non-small cell lung cancer (NSCLC) patients treated with first-generation epidermal growth factor receptor-tyrosine kinase inhibitor (EGFR-TKI) almost always acquire resistance, and the development of novel techniques analyzing circulating tumor DNA (ctDNA) have made it possible for liquid biopsy to detect genetic alterations from limited amount of DNA with less invasiveness. While a large amount of patients with EGFR exon 21 p.Thr790 Met (T790M) benefited from osimertinib treatment, acquired resistance to osimertinb has subsequently become a growing challenge.

**Methods:**

We performed tissue and liquid rebiopsy on 50 patients with EGFR-mutant NSCLC who acquired resistance to first-generation EGFR-TKIs. Plasma samples underwent droplet digital PCR (ddPCR) and next-generation sequencing (NGS) examinations. Corresponding tissue samples underwent NGS and Cobas^®^ EGFR Mutation Test v2 (Cobas) examinations.

**Results:**

Of the 50 patients evaluated, the mutation detection rates of liquid biopsy group and tissue biopsy group demonstrated no significant differences (41/48, 85.4% vs. 44/48, 91.7%; OR=0.53, 95% CI=0.15 to 1.95). Overall concordance, defined as the proportion of patients for whom at least one identical genomic alteration was identified in both tissue and plasma, was 78.3% (36/46, 95% CI=0.39 to 2.69). Moreover, our results showed that almost half of the patients (46%, 23/50) resistant to first-generation EGFR-TKI harbored p.Thr790 Met (T790M) mutation. 82.6% (19/23) of the T790M positive patients were analyzed by liquid biopsy and 60.9% (14/23) by tumor tissue sequencing. Meanwhile, a wide range of uncommon mutations was detected, and novel mechanisms of osimertinib resistance were discovered. In addition, 16.7% (2/12) of the T790M positive patients with either TP53 R237C or KRAS G12V failed to benefit from the subsequent osimertinib treatment.

**Conclusion:**

Our results emphasized that liquid biopsy is applicable to analyze the drug resistance mechanisms of NSCLC patients treated with EGFR-TKIs. Moreover, we discovered two uncommon mutations, TP53 R273C and KRAS G12V, which attenuates the effectiveness of osimertinib.

## Introduction

With advances in genomic research and targeted therapy, the therapeutic strategies and prognosis of patients with NSCLC, especially EGFR-mutated lung adenocarcinoma, have been greatly improved (1). Even though EGFR-targeted therapy has become the standard treatment in advanced EGFR-positive NSCLC, most patients eventually develop acquired resistance to first-generation EGFR-TKIs after 12–24 months of treatment (2–4). Among many reasons leading to acquired resistance, the T790M mutation in an exon of the EGFR gene accounts for more than half of the cases, followed by other mechanisms, including human epidermal growth factor receptor-2 (HER2) amplification, phenotypic transformation, and mesenchymal epithelial transition proto-oncogene (MET) amplification (5–10). Hence, genome sequencing is required to determine the presence of the T790M mutation before the application of the third-generation EGFR-TKI osimertinib, which targets T790M (11, 12). Moreover, there are still patients with the T790M mutation who do not respond or partially respond to osimertinib, suggesting that additional resistance mechanisms may decrease the efficacy of osimertinib (13–15). A repeatable assay to comprehensively detect and monitor somatic alterations with high sensitivity during EGFR-TKI treatment is therefore required.

The emergence of novel techniques has allowed the discovery of multiple genetic mutations in malignant tumors (16). Tissue-based analyses have been widely used to detect driver or resistance mutations in NSCLC, providing clinicians with valuable therapeutic insights. However, it also has evident limitations considering the invasiveness of tumor biopsy, and the insufficient size of specimens obtained might neglect the spatial and temporal tumor heterogeneity (17–19). Therefore, liquid biopsy caught our attention. Liquid biopsy is a diagnostic method that can repeatedly collect ctDNAs from multiple tumor deposits with minimal invasiveness (20, 21). ctDNAs harbor tumor-specific sequence alterations from tumor cells derived from primary tumors and metastatic lesions, allowing a more comprehensive analysis of the tumor genome than tissue DNA from a single site (22–24). While tissue biopsy is still irreplaceable in diagnosing tumor malignancy, we intend to investigate whether liquid biopsy could be conducted to detect EGFR-TKI resistance mechanisms in NSCLC patients and hopefully save patients from undue suffering.

Despite the increasing utilization of plasma analyses in guiding clinical decisions, few studies have evaluated the efficiency of different approaches. NGS has been recognized as an efficient and reliable approach to detect multiple genetic mutations simultaneously (25, 26). On the other hand, ddPCR features extremely high sensitivity with a relatively narrow detection range (27, 28). Such advantages and limitations can be influential for the determination of subsequent treatment. In the present study, we used ddPCR and NGS sequencing to analyze plasma samples, and NGS and Cobas sequencing to analyze tissue samples to identify somatic mutations in EGFR-positive NSCLC patients during disease progression. We found a similar detection rate and a high correspondence between liquid biopsy and conventional tissue biopsy, while ddPCR and NGS each have advantages. Meanwhile, our results showed a wide spectrum of gene mutations, and two uncommon EGFR mutations were identified related to osimertinib resistance. Herein, our study emphasized the utility of liquid biopsy and might uncover two novel molecular mechanism of resistance.

## Materials and Methods

### Study Population and Inclusion Criteria

This was a multi-centre study performed at the Department of Respiratory and Critical Care Medicine in 15 hospitals located in Jiangsu Province and Anhui Province in East China. The inclusion criteria were as follows (1): Patients histologically diagnosed with stage IIIB or IV lung adenocarcinoma. (2) EGFR mutations that were previously detected and treated with first-generation EGFR-TKIs, including gefitinib, erlotinib, and icotinib. (3) Presently graded progressive disease (PD) after first-line EGFR-TKI treatment. (4) Availability of rebiopsied tumor tissue or liquid biopsy samples for analysis. A total of 50 patients with advanced NSCLC were recruited from 01 February 2018 to 01 June 2019. Twenty-seven (54%) were male, and 23 (46%) were female, with a median age of 65 years (range, 44–84 years). Two patients had plasma samples only, and two patients underwent tissue rebiopsy only, while all the other patients’ tissue and paired plasma samples were collected within three days, and detections were finished within seven days. This study was approved by the institutional review board of the First Affiliated Hospital of Soochow University (No. 2018013). All patients enrolled in this study signed an informed consent form.

### Specimen Collection and DNA Extraction

The tumor tissues and/or paired peripheral blood samples (8 ml) from each patient were collected within the scope of routine diagnostic procedures and were available in all patients. Four to eight sections of fresh tissue immersed in formalin (5-mm thickness) or formalin-fixed, paraffin-embedded (FFPE) diagnostic tumor tissue samples were used for tissue DNA extraction. The DNA from tumor tissues was isolated from the sample using the QIAamp DNA Mini Kit (Qiagen, Hilden, Germany), and the DNA from FFPE slides was isolated by using a QIAamp DNA FFPE Tissue Kit (Qiagen, Hilden, Germany). Both procedures were performed following the specific manufacturer’s instructions. Peripheral blood samples were collected in cell-free DNA BCT^®^ tubes (Streck, La Vista, Nebraska, U.S.) and processed within 6 h. The samples were centrifuged at 3,000 rpm for 10 min at 4°C and then centrifuged for a second time at 16,000 g for 10 min at 4°C. Plasma was then separated and stored at −80°C until cfDNA extraction. Circulating cell-free DNA was extracted from at least 4 ml of plasma using a QIAamp Circulating Nucleic Acid kit (Qiagen, Hilden, Germany) according to the manufacturer’s protocol. All the procedures for the molecular analysis were performed following the specific manufacturer’s instructions. Mutations were then detected using the eluted DNA samples.

### Mutation Detection

In a subset of cases (n = 50), ddPCR was carried out for hotspot mutations in EGFR using the QX100 Droplet Digital PCR System (Bio-Rad, Hercules, CA) at Shanghai Yuanqi Bio-pharmaceutical Company. Positivity was defined as the fractional abundance of ≥0.044% for the plasma samples. NGS was carried out using the Ion AmpliSeqTM Cancer Hotspot Panel v2 on the Ion Proton sequencer (ThermoFisher Scientific) at Shanghai Singlera Genomics. NGS data were analyzed using Torrent Suite software (version 5.0.2). Results that above the detection limitation of 0.1% were considered positive. The real-time PCR Cobas^®^ EGFR Mutation Test Kit (Roche Diagnostics) uses a pool of primers allowing for a target size from 85 to 155 bp, which was divided into three different mixes for each sample and control. The Cobas assay was run using the Cobas z480 thermocycler (Roche Diagnostics) at Shanghai Di-an Diagnosis Technology Company and all the positive results were included.

### Statistical Analysis

The chi-square test was used to calculate the concordance between mutation detection rates of plasma and tissue samples. The Pearson correlation coefficient was used to assess the correlation of variant frequencies between the assays. Multivariable logistic regression was performed to evaluate the association between clinical characteristics and ctDNA detection rate. The cut-off date used for therapy outcome analysis was 31 October 2019. Data were analyzed by SPSS Statistics version 25.0 (IBM Corporation, NY, USA) and SPSS 17.0 software (SPSS, Chicago, IL). All P values are two-sided, and confidence intervals are at the 95% level, with statistical significance defined as P < 0.05.

## Results

### Mutation Detection in Oncogenes After Resistance

We used four different approaches to detect mutations among the 50 patients with advanced EGFR-mutant lung cancer who acquired resistance to EGFR-TKIs, including ddPCR and NGS assays performed in plasma samples and NGS and Cobas assays performed in tissue samples. A total of 50 patients from 15 hospitals, including 27 males and 23 females, were recruited for our investigation. The clinical characteristics of the patients are summarized in **Table 1**. All patients were initially diagnosed with lung adenocarcinoma with an EGFR mutation and received first-generation EGFR-TKI treatment. According to the National Comprehensive Cancer Network (NCCN) guidelines for NSCLC, it is recommended to perform routine mutation tests for EGFR, BRAF, ERBB2, and MET as well as rearrangements in ALK, ROS1, and RET in all patients diagnosed with advanced NSCLC. Here, we performed the NGS panel containing 12 genes covering all the mutations mentioned above, and the ddPCR and Cobas assays each had their respective deficiencies, which are described in **Table 2**.

**Table 1:** Clinical characteristics of enrolled NSCLC patients with EGFR mutations (n = 50).

Clinical characteristics	Number of patients (%)
Gender	
Man	27 (54)
Women	23 (46)
Age, median (range)	65 (44–84)
Histological type	
Adenocarcinoma	50 (100)
Cancer stage	
IIIb	12 (24)
IV	38 (76)
EGFR-activating mutation	
Exon 19 deletion	19 (38)
Exon 21 L858R mutation	18 (36)
Exon 18 G719X mutation	1 (2)
Unknown	12 (24)
Previous EGFR-TKI therapy	
Gefitinib	28 (56)
Erlotinib	6 (12)
Icotinib	16 (32)
Sample type upon PD	
Plasma only	2 (4)
Tissue only	2 (4)
Plasma and tissue	46 (92)

**Table 2:** Detectable mutations by assay.

Gene	Exon	Mutation	Plasma		Tissue	
			ddPCR	NGS-plasma	NGS-tissue	Cobas^®^
EGFR	18	G719X	√	√	√	√
	19	19-del	√	√	√	√
	20	T790M	√	√	√	√
	20	Insert		√	√	√
	21	L858R	√	√	√	√
		Others		√	√	
ALK				√	√	
ALK Fusion	20			√	√	
BRAF				√	√	
ERBB2				√	√	
FGFR1				√	√	
MET				√	√	
NRAS				√	√	
KRAS				√	√	
PIK3CA				√	√	
RET Fusion	10			√	√	
	11			√	√	
	12			√	√	
ROS1 Fusion	32			√	√	
	33			√	√	
	34			√	√	
	35			√	√	
	36			√	√	
TP53				√	√	

**Figure 1:**
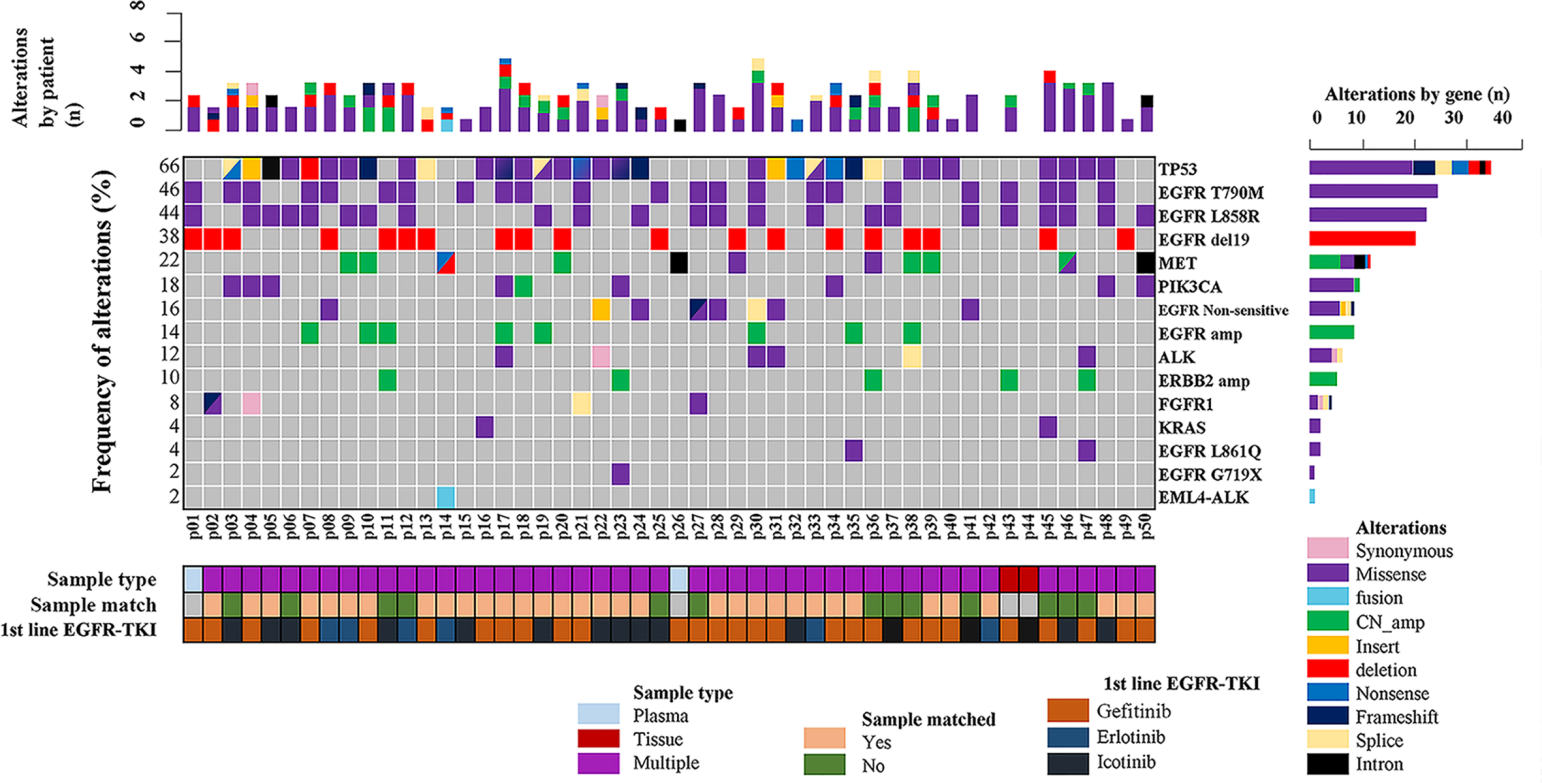
Summary of putative resistance mechanisms to first-generation EGFR-TKIs identified in 50 patients.

In the present study, 48 screened patients provided matched tumor tissue and plasma samples. Among the genotyping results obtained from 96 samples in 50 patients, a total of 48 ddPCR results and 46 NGS results were obtained from 48 plasma samples (2 plasma samples received no NGS results due to quality failure), while 48 NGS results and Cobas results were obtained from tumor tissue samples. A total of 46 out of 50 (92%) patients underwent both liquid biopsy and tissue rebiopsy for multimethod genotype sequencing after tumor progression. The distribution of the gene mutations classified by patients and alterations is shown in **Figure 1**.

In total, somatic mutations were found in 96% (48/50) of the patients. NGS and ddPCR detected somatic mutations in 82.6% (38/46) and 70.8% (34/48) of the plasma samples, while NGS and Cobas detected gene alterations in 93.6% (44/47) and 69.4% (25/36) of the tissue samples, respectively. Thus, the mutation detection rate was slightly lower in samples of plasma than in tissue with no significant difference (41/48, 85.4% vs. 44/48, 91.7%; OR = 0.53, 95% CI = 0.15 to 1.95, P = 0.336), and NGS was the most sensitive among all the approaches. The overall concordance between plasma and tissue sequencing results, defined as the proportion of patients who carried at least one matching result between each of the two methods in tissue and plasma examining, was 78.3% (36/46, 95% CI = 0.39 to 2.69).

Multivariable logistic regression on ctDNA detection rate and clinical factors such as age, sex, and disease stage did not identify any statistically significant variables that predicted the detection of plasma ctDNA.

### Resistant Mechanisms to First-Generation EGFR-TKI Treatment

**Figure 2:**
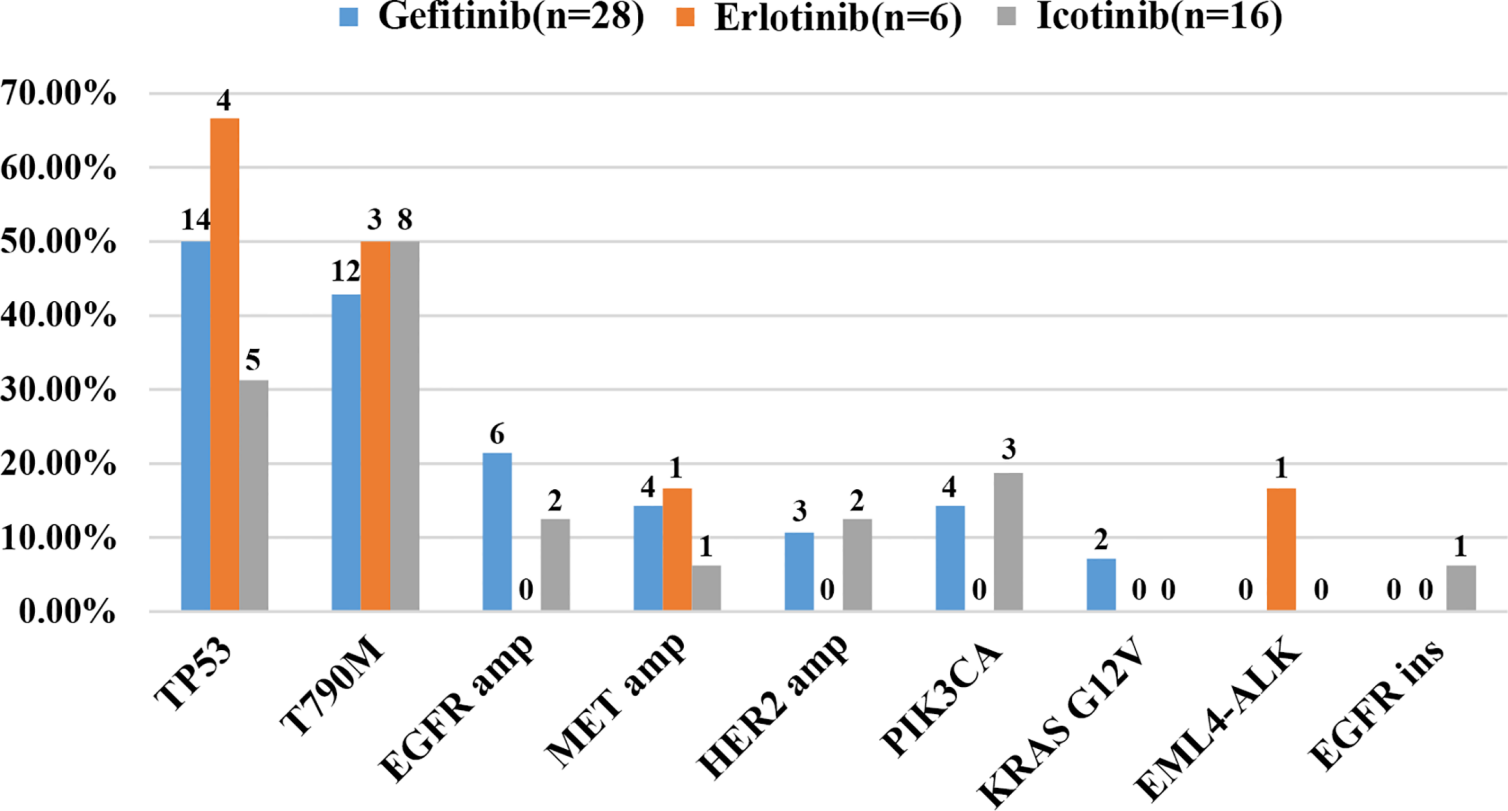
Cases of potential resistance mechanisms detected in patients sorted by different EGFR-TKIs.

As demonstrated in **Figure 1**, TP53 was the most frequently mutated gene (33/50, 66%), followed by EGFR T790M (23/50, 46%). Sensitive EGFR mutations, including ex19del, L858R, G719X, and L816Q alterations, were found in 82% (41/50) of the patients. In addition, resistance mechanisms to EGFR-TKIs are known to be heterogeneous, and multiple aberrations may be present simultaneously, which is consistent with our findings (18, 29, 30). Ninety percent (45/50) of the patients bore more than one genomic alteration. During first-line treatment, 28 of the patients (56%) received gefitinib, 6 of them (12%) received erlotinib, and the remaining 16 (32%) were treated with icotinib. Hence the nine most frequently detected resistant mutations that might have clinical significance were sorted by different subsets of first-generation TKIs in **Figure 2**, and the pattern of resistance mechanisms to gefitinib, erlotinib, and icotinib appeared to differ.

The prevalence of the TP53 mutation was still the highest and was observed in 46% of the patients (23 of 50), especially among patients treated with gefitinib and erlotinib. Interestingly, the T790M mutation, known to be the most common mechanism of acquired resistance to gefitinib, was first identified in the current study. It was also detected in 46% of all patients (23/50) and 43% of patients treated with gefitinib (12/28). EGFR amplification (16%, 8/50) and MET amplification (12%, 6/50) were also observed. Apart from these differences, there were no significant differences in the prevalence of other resistance mechanisms between the three groups. Despite the limited number of samples analyzed, these results might reflect the heterogeneity of TKI resistance.

### Detection Rate of the T790M Mutation

**Figure 3:**
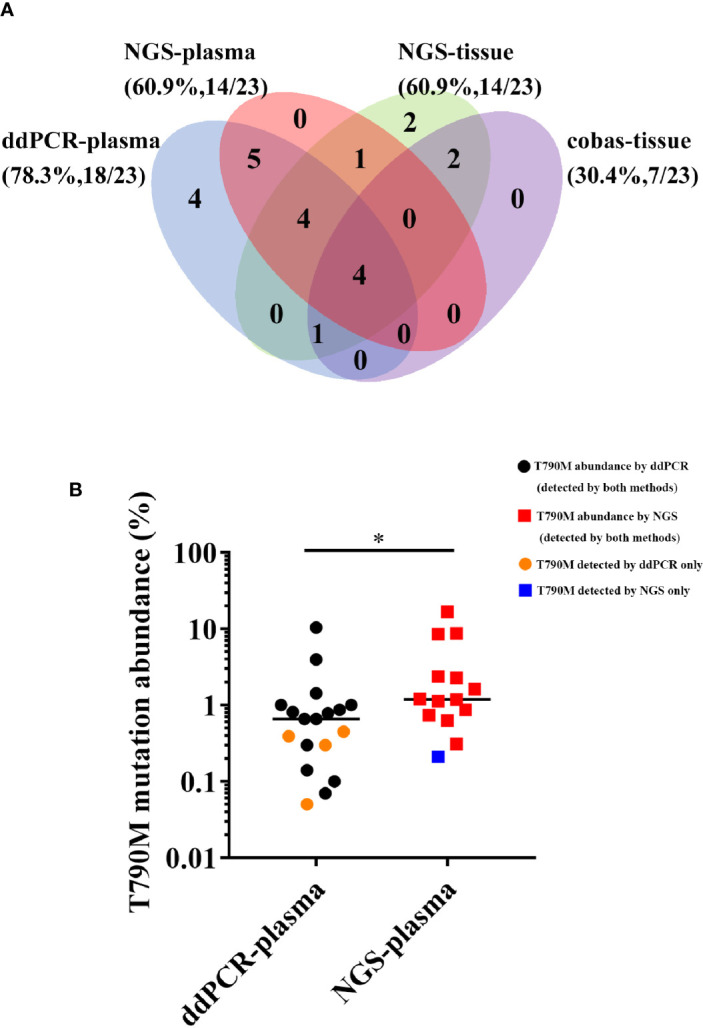
**(A)** Comparison of T790M detection among four different approaches. **(B)** Comparison of the T790M abundance detected by ddPCR and NGS in plasma samples. (*P < 0.05).

We next investigated the detection rate of T790M, the gene alteration most related to second-line therapy after EGFR-TKI resistance, among four different methods. The results are shown in the form of a Venn diagram in **Figure 3A**. Among all the T790M-positive patients, 19 of 23 were detected in plasma samples, while 14 of 23 were detected in tissue samples. The concordance between plasma and tissue NGS genotyping for the T790M mutation was 50% (9/18), and the detection rate of T790M was the same in NGS-plasma samples and in NGS-tissue samples (60.9%). Furthermore, ddPCR and NGS detected EGFR T790M mutations in 78.3% and 60.9% of plasma samples respectively, and the detection concordance between NGS-plasma and ddPCR is 68.4% (13/19). We further determined that T790M abundance detected by ddPCR was significantly lower than NGS in **Figure 3B**. Combined with the information in **Table 2**, we concluded that NGS is a more comprehensive approach with a relatively satisfactory detection rate. On the other hand, ddPCR is a more sensitive test, especially regarding the T790M mutation, despite its comparatively small examination range.

### Potential Resistance Mechanisms to Osimertinib Treatment

According to the follow-up investigation, 9 of the 23 T790M-positive patients withdrawn from EGFR-TKIs treatment and chose chemotherapy of palliative treatment due to the relatively high expense of osimertinib. The other two patients were ruled out because their T790M mutation was detected by only one approach that could lead to low credibility. Thus, we analyzed 12 cases that received third-generation EGFR-TKI osimertinib treatment, as shown in **Table 3**. Among the 12 patients, 10 showed stable disease or reduced tumor size by computerized tomography (CT) scanning, while 2 of them exhibited disease progression. Moreover, most of the patients (9/12, 75.0%) showed more than one genetic mutation, including T790M combined with a TP53 point mutation (2/12, 16.7%), a PIK3CA point mutation (3/12, 25.0%), or even a triple mutation like EGFR L858R/MET/PIK3CA. Based on the assumption that coexisting mutations may be related to the mechanism underlying the development of EGFR-TKI resistance, we further investigated two patients, No. 17 and No. 45, who progressed on osimertinib and found several seldom studied point mutations.

**Table 3:** Response of patients with T790M mutation to osimertinib.

ID	Resistance mutation	PFS to first-generation EGFR-TKIs (month)	Response to therapy
03	EGFR T790M^L^, PIK3CA R88Q^L^	30	Effective
04	EGFR T790M^L,T^, PIK3CA E545K^L,T^	12	Effective
08	EGFR T790M^L,T^, TP53 C124Ter ^T^	14	Effective
11	EGFR T790M^L^, EGFR Amp^L^, ERBB2 Amp ^T^	13	Effective
15	EGFR T790M^L^	4	Effective
17	EGFR T790M^L,T^, EGFR Amp ^T^, TP53 R273C^L^	35	Progression
18	EGFR T790M^L,T^, PIK3CA Amp ^T^	35	Effective
25	EGFR T790M^L,T^	52	Effective
30	EGFR T790M^L,T^, EGFR Amp ^T^, TP53 D281G ^T^	12	Effective
33	EGFR T790M^L,T^, TP53 c.559+1G>A ^T^, TP53 R249S^L^	19	Effective
41	EGFR T790M ^T^	7	Effective
45	EGFR T790M ^T^, KRAS G12V^L^, TP53 G244D ^T^	5	Progression

L, detected by liquid biopsy; T, detected by tissue biopsy, PFS, progression-free survival.

**Figure 4:**
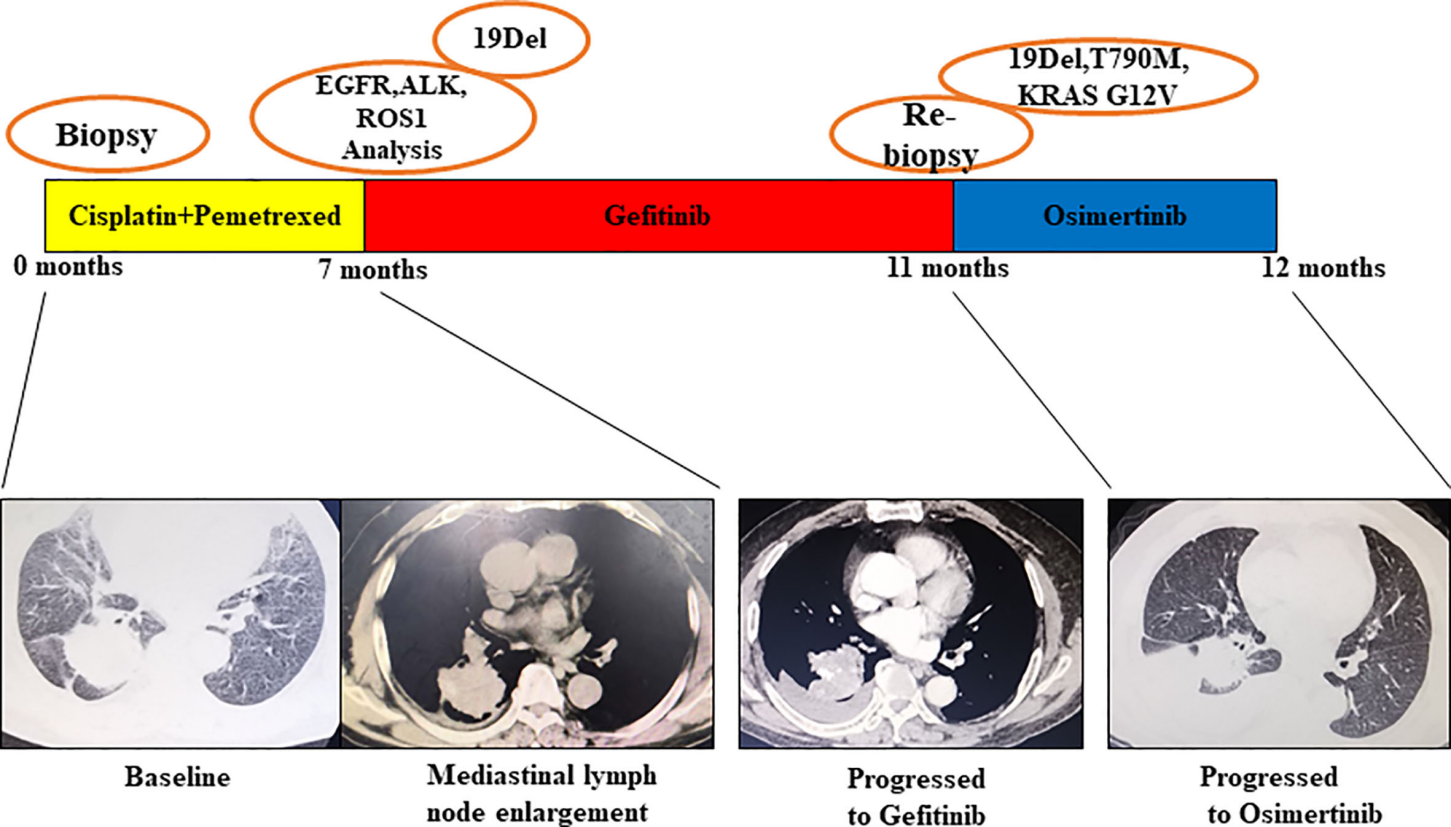
Illustration of the genotype data and treatment received by patient No. 17 along with representative CT scans at the time points indicate.

Patient No. 17, a 75-year-old female, never smoker, was diagnosed with right lung adenocarcinoma positive for EGFR ex19del. The patient was initially treated with first-generation EGFR-TKI gefitinib since EGFR exon 19 deletion was detected upon diagnosis biopsy. However, disease progressed 32 months after occurrence, as shown in **Figure 4**. A rebiopsy revealed EGFR T790M, TP53 R273C mutation, and EGFR amplification, with persistence of EGFR ex19del. Hence, second-line treatment with osimertinib was carried out. Unfortunately, disease progression occurred 3 months later, treatment with osimertinib was discontinued. Considering that other patients harboring EGFR T790M and EGFR amplification have shown sensitivity to osimertinib treatment (31), we identified the TP53 R273C mutation to promote resistance to osimertinib.

**Figure 5:**
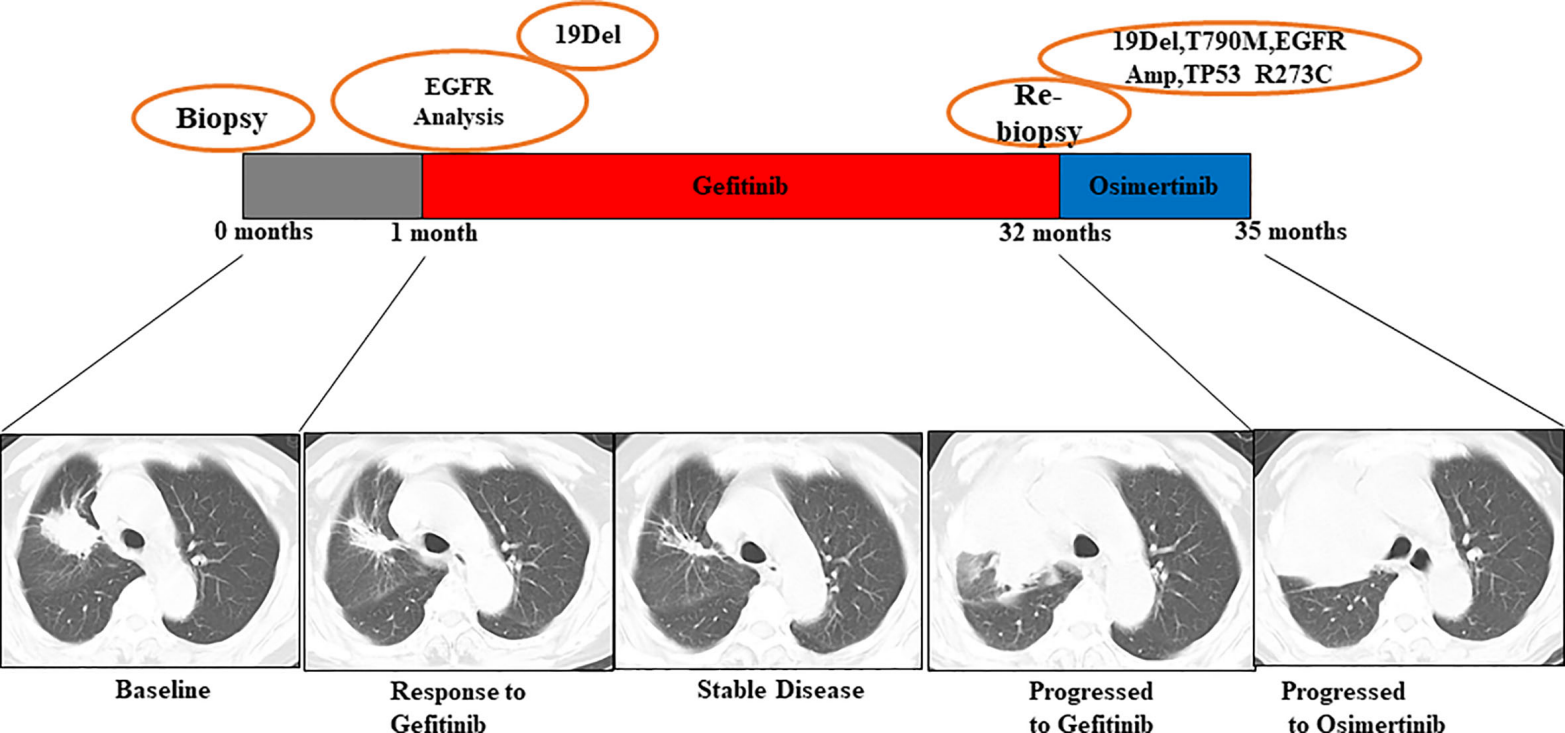
Illustration of the genotype data and treatment received by patient No. 45 along with representative CT scans at the time points indicated.

Patient No. 45, a 72-year-old male, presented with uncontrollable cough, after which he was diagnosed with an EGFR-mutated ex19del right lung adenocarcinoma. First, chemotherapy with cisplatin and pemetrexed was used for a total of 7 months until mediastinal lymph node enlargement was observed. Gefitinib was then administered, being optimally tolerated at the beginning. However, after 4 months, gefitinib was discontinued because of disease progression, as shown in **Figure 5**. The subsequent rebiopsy results confirmed ex19del and revealed the emergence of EGFR T790M, KRAS G12V, and TP53 G244D mutations. The patient then commenced a new treatment with osimertinib. However, his condition rapidly declined, and platinum-based combination chemotherapy was initiated. Since there is currently no report about TP53 G244D mutation-induced TKI resistance, it is suggested that the KRAS G12V mutation may prompt resistance to third-generation EGFR-TKIs.

## Discussion

Acquired resistance to EGFR-TKIs has become an important issue for NSCLC patients with EGFR activating mutations and is mostly due to secondary mutations such as T790M (5, 6). Given that former studies have shown intra-patient heterogeneity of resistant mechanisms (8, 9, 32), rebiopsies were requested at disease progression in the detection of putative resistant mutations. However, traditional bronchoscopy tissue biopsy has limitations, such as invasiveness. More importantly, tissue biopsy can only analyze one single tumor deposit at a time and requires a sufficient amount of tissue to perform all the assays (19), which would likely underestimate the temporal and spatial heterogeneity in resistance mutations (17, 18). Hence, a novel method for detecting EGFR-TKI-resistant mutations with high sensitivity and accuracy is urgently required for clinical practice. Here, we aimed to determine the most suitable rebiopsy assay for patients with advanced NSCLC resistant to first-line EGFR-TKIs.

Liquid biopsy caught our attention in that it can simultaneously capture and detect mutations in multiple tumor deposits with minimum invasiveness. It can also detect different types of gene alterations, including single nucleotide variants, copy number variants, deletions, and fusion genes. Thus, analysis of ctDNA can be conducted repeatedly throughout the whole course of treatment to monitor genomic alteration (22, 33–35). In the present study, we compared the efficiency of four different methods of genomic sequencing for the first time. We used NGS and Cobas sequencing for tissue samples and detected somatic mutations in 91.7% (44/48) of samples. NGS and ddPCR were used for plasma samples, and the respective detection rate was 85.4% (41/48). The concordance between tissue and plasma genotyping was 78.3% (36/46), indicating that there is no significant difference between their detection rates. Considering the advantages mentioned before, our findings suggest that liquid biopsy is more applicable to EGFR-TKI resistance detection than tissue biopsy.

Subsequently, we sought to identify the difference between two assays of liquid biopsy, ddPCR and NGS. In performing ctDNA analysis, we observed a high frequency of molecular heterogeneity in resistance mechanisms following treatment of NSCLC patients with first-generation EGFR-TKIs. NGS has the advantage of comprehensively detecting multiple gene alterations (36). It is capable of testing thousands of genes or even the whole genome with a relatively low DNA input (26, 37). On the other hand, ddPCR showed the highest sensitivity in detecting certain gene mutations that guide second-line treatment, such as T790M (28). Its detection rate of T790M is higher than that of NGS (78.3% vs. 60.9%), and ddPCR can identify T790M at a significantly lower abundance than NGS. In summary, NGS can provide a comprehensive view of the genome, while ddPCR is applicable to detecting specific mutations guiding clinical practice.

After we identified 12 patients with the T790M mutation by different assays, osimertinib treatment was initiated. Previous studies have confirmed the effectiveness of the third-generation TKI osimertinib for patients with acquired T790M mutations upon disease progression (38–40). However, the duration of response varies even for patients who initially respond to osimertinib, since heterogeneity has long been an obstacle in targeted treatment (30). Recent researched have shown spatial temporal heterogeneity among lung cancer, and the emergence of concurrent mutations such as MET amplification or other driver oncogene before or during pharmacologic intervention can also induce osimertinib resistance (18, 29, 41). Our study here revealed two patients who demonstrated no response to osimertinib treatment. Given that multiple gene alterations were detected in our study and that prior studies have observed intrapatient heterogeneity of resistance mechanisms in NSCLC patients with EGFR mutations, we hypothesized that the mutations coexisting with T790M may impact the efficacy of treatment with third-generation EGFR-TKIs. Moreover, concurrent TP53 alterations have been considered to be related to a lower likelihood of responding to EGFR-TKIs and a shorter PFS (11, 42, 43), and TP53 R273 has been confirmed able to reduce cell apoptosis which might be the mechanism underlying R273C induced osimertinib resistance (44, 45). Studies have suggested that KRAS mutations are associated with decreased responsiveness to EGFR-TKIS in NSCLC (16, 46), probably because KRAS mutation can lead to the activation of downstream RAF-MEK-ERK pathway (47). KRAS G12V that we concerned was found more likely to induce EGFR-TKI resistance by regulating MEK/ERK than PI3K/AKT pathway (48). Thus, we identified two candidate mutations in patient No. 17 and No. 45, TP53 R273C and KRAS G12V, that may have antagonistic effects on the efficacy of osimertinib. Collectively, these findings underlined that genomic results of liquid biopsy can be indicative of the clinical response to second-line EGFR-TKI treatment, such as osimertinib.

There are still limitations to our retrospective study. First, it included a relatively small number of patients. Second, analysis of plasma DNA at baseline was not performed. Finally, the efficacy of subsequent treatment with osimertinib according to genetic profile was not monitored in our study.

Our study showed that liquid biopsy is sufficient to identify somatic mutations and resistance mechanisms at disease progression during treatment with first-generation EGFR-TKIs compared to tissue biopsy. Different approaches of ctDNA analysis have their respective advantages and limitations. Hence, a combination of multiple approaches can offer patients the best therapeutic options.

## Data Availability Statement

The original contributions presented in the study are included in the article/**Supplementary Material**. Further inquiries can be directed to the corresponding authors.

## Ethics Statement

The studies involving human participants were reviewed and approved by the First Affiliated Hospital of Soochow University. The patients/participants provided their written informed consent to participate in this study.

## Author Contributions

J-AH and ZL designed the experiments. YF, AW, and JZ analyzed the data and wrote the manuscript. WF, MS, XX, HZ, LC, JF, XL, XZ, WX, ZZ, GM, JW, TZ, DZ, and HF provided tissue and/or plasma samples and clinical data. All authors contributed to the article and approved the submitted version.

## Funding

This work was supported by the Suzhou Key Laboratory for Respiratory Medicine (grant number SZS201617), the Clinical Medical Center of Suzhou (grant number Szzx201502), the Jiangsu Provincial Key Medical Discipline (grant number ZDXKB2016007), and the Clinical Key Specialty Project of China.

## Conflict of Interest

The authors declare that the research was conducted in the absence of any commercial or financial relationships that could be construed as a potential conflict of interest.
