# Corrigendum: Advanced NSCLC patients with EGFR T790M harbouring TP53 R273C or KRAS G12V cannot benefit from osimertinib based on a clinical multicentre study by tissue and liquid biopsy

**DOI:** 10.3389/fonc.2023.1236311

**Published:** 2023-06-27

**Authors:** Yulong Fu, Anqi Wang, Jieqi Zhou, Wei Feng, Minhua Shi, Xiao Xu, Hongqing Zhao, Liming Cai, Jian Feng, Xuedong Lv, Xiaodong Zhang, Wenjing Xu, Zhengrong Zhang, Guoer Ma, Jian Wang, Tong Zhou, Dahai Zhao, Haohui Fang, Zeyi Liu, Jian-an Huang

**Affiliations:** ^1^ Department of Pulmonary and Critical Care Medicine, The First Affiliated Hospital of Soochow University, Suzhou, China; ^2^ Suzhou Key Laboratory for Respiratory Diseases, Suzhou, China; ^3^ Department of Respiratory Medicine, The Second Affiliated Hospital of Soochow University, Suzhou, China; ^4^ Department of Respiratory Medicine, Affiliated Suzhou Hospital of Nanjing Medical University, Suzhou, China; ^5^ Department of Respirology, Nanjing Medical University Affiliated Wuxi Second Hospital, Wuxi, China; ^6^ Department of Respiratory Medicine, Affiliated Hospital of Jiangnan University, Wuxi, China; ^7^ Department of Respiratory Medicine, Affiliated Hospital of Nantong University, Nantong, China; ^8^ Department of Respiratory Medicine, The Second Affiliated Hospital of Nantong University, Nantong, China; ^9^ Department of Medical Oncology, Nantong Tumor Hospital, Nantong, China; ^10^ Departments of Respiratory Medicine, Northern Jiangsu People’s Hospital, Clinical Medical College of Yangzhou University, Yangzhou, China; ^11^ Department of Respiratory Medicine, First People’s Hospital of Yangzhou City, Yangzhou, China; ^12^ Department of Respiratory Medicine, Affiliated Hospital of Jiangsu University, Zhenjiang, China; ^13^ Department of Respiratory Medicine, Zhenjiang First People’s Hospital, Zhenjiang, China; ^14^ Department of Oncology, Changzhou Cancer Hospital Affiliated to Soochow University, Changzhou, China; ^15^ Department of Respiratory and Critical Care Medicine, The Second Hospital of Anhui Medical University, Hefei, China; ^16^ Department of Respiratory Medicine, Anhui Chest Hospital, Hefei, China; ^17^ Institute of Respiratory Diseases, Soochow University, Suzhou, China

**Keywords:** non-small cell lung cancer, liquid biopsy, gene sequencing, epidermal growth factor receptor-tyrosine kinase inhibitor (EGFR-TKI), drug resistance

**Error in Figure/Table Legend**

In the published article, there was an error in the legend for Figure 4 as published. The number of the patient was wrong. The corrected legend appears below.

Illustration of the genotype data and treatment received by patient No. 45 along with representative CT scans at the time points indicated.

**Error in Figure/Table Legend**

In the published article, there was an error in the legend for Figure 5 as published. The number of the patient was wrong. The corrected legend appears below.

Illustration of the genotype data and treatment received by patient No. 17 along with representative CT scans at the time points indicate.

**Text Correction**

In the published article, there was an error. The number of patients from different sexes were wrong.

A correction has been made to 3. RESULTS, *3.1 Mutation detection in oncogenes after resistance*, Paragraph 1. This sentence previously stated:

“A total of 50 patients from 15 hospitals, including 27 females and 23 males, were recruited for our investigation.”

The corrected sentence appears below:

“A total of 50 patients from 15 hospitals, including 27 males and 23 females, were recruited for our investigation.”

**Text Correction**

In the published article, there was an error. The description of patient No.17 was inconsistent with the content of the Figures.

A correction has been made to 3. RESULTS, *3.4 Potential resistance mechanisms to osimertinib treatment*, Paragraph 2. This sentence previously stated:

“First, chemotherapy with cisplatin and pemetrexed was used for a total of 7 months until mediastinal lymph node enlargement was observed. Gefitinib was then administered, being optimally tolerated at the beginning. However, after 4 months, gefitinib was discontinued because of disease progression, as shown in Figure 4. A rebiopsy revealed EGFR T790M, TP53 R273C mutation, and EGFR amplification, with persistence of EGFR ex19del. The patient then commenced a new treatment with osimertinib. However, her condition rapidly declined, and she died 1 month later.”

The corrected sentence appears below:

“The patient was initially treated with first-generation EGFR-TKI gefitinib since EGFR exon 19 deletion was detected upon diagnosis biopsy. However, disease progressed 32 months after occurrence, as shown in Figure 5. A rebiopsy revealed EGFR T790M, TP53 R273C mutation, and EGFR amplification, with persistence of EGFR ex19del. Hence, second-line treatment with osimertinib was carried out. Unfortunately, disease progression occurred 3 months later, treatment with osimertinib was discontinued.”

**Text Correction**

In the published article, there was an error. The description of patient No.45 were inconsistent with the content of the Figures.

A correction has been made to 3. RESULTS, *3.4 Potential resistance mechanisms to osimertinib treatment*, Paragraph 3. This sentence previously stated:

“The patient was initially treated with first-generation EGFR-TKI gefitinib since EGFR exon 19 deletion was detected upon diagnosis biopsy. However, disease progression occurred 32 months after treatment initiation, as shown in Figure 5. The subsequent rebiopsy results confirmed ex19del and revealed the emergence of EGFR T790M, KRAS G12V, and TP53 G244D mutations. Hence, second-line treatment with osimertinib was carried out. Unfortunately, disease progression occurred 3 months later, treatment with osimertinib was discontinued,”

The corrected sentence appears below:

“First, chemotherapy with cisplatin and pemetrexed was used for a total of 7 months until mediastinal lymph node enlargement was observed. Gefitinib was then administered, being optimally tolerated at the beginning. However, after 4 months, gefitinib was discontinued because of disease progression, as shown in Figure 4. The subsequent rebiopsy results confirmed ex19del and revealed the emergence of EGFR T790M, KRAS G12V, and TP53 G244D mutations. The patient then commenced a new treatment with osimertinib. However, his condition rapidly declined,”

The authors apologize for these errors and state that this does not change the scientific conclusions of the article in any way. The original article has been updated.

