# Supplementary material for: Advanced NSCLC Patients With EGFR T790M Harboring TP53 R273C or KRAS G12V Cannot Benefit From Osimertinib Based on a Clinical Multicentre Study by Tissue and Liquid Biopsy
Source: Front Oncol. 2021 Feb 24;11:621992. doi: 10.3389/fonc.2021.621992 (PMC7943858; doi:10.3389/fonc.2021.621992)
Supplement: Supplementary file 1 [file Table_1.docx]

Supplementary Table 1. Summary of all the mutations detected in each patient including variant allele frequency (VAF)

| ID | Plasma | | Tissue | |
| --- | --- | --- | --- | --- |
|  | ddPCR | NGS | NGS | Cobas |
| 1 | 19Del(0.39%), T790M(1.0%) | Invalid | Invalid | Invalid |
| 2 | 19Del(7.66%) | 19 Glu746_Ala750del(2.92%), FGFR1 11 p.Ala462ValfsTer3 29.68%, FGFR1 11 Val460Leu 1.02% | Invalid | 19Del |
| 3 | 19Del(7.23%), T790M(3.97%) | 19 Glu746_Ala750del(14.78%), 20 T790M(8.49%), PIK3CA 2 Arg88Gln(7.36%), TP53 c.97-1G>A 5.75% | 19 Glu746_Ala750Del(3.01%), TP53 9 Gln331Ter 11.76% | Invalid |
| 4 | L858R(0.61%), T790M(0.81%) | 21 L858R(1.51%), 20 T790M(0.87%), PIK3CA 10 Glu545Lys(0.77%), FGFR1 6 c.543C>T 1.36% | TP53 10 Gly334_Arg335dup 13.27%, 21 L858R(13.64%), 20 T790M(6.98%), PIK3CA 10 Glu545Lys(8.16%) | Invalid |
| 5 | L858R(0.43%) | 21 L858R(1.72%) | TP53 c.560-24_560-1delTGATTCCTCACTGATTGCTCTTAG 23.07%, 21 L858R(39.20%) | L858R |
| 6 | No EGFR mutant | No EGFR mutant | 21 L858R 10.99%, PIK3CA 21 His1047Arg （8.60%), TP53 8 (Glu285Lys 9.47%) | Invalid |
| 7 | L858R(21.4%), T790M(0.66%) | 21 L858R(40.44%), 20 T790M(1.62%), TP53 5 Pro142_Val143del 1.82% | TP53 5 Pro142_Val143del 82.61%, 21 L858R (96.35%), EGFR Amp 14.65, EGFR 20 Thr790Met 1.4% | L858R, T790M |
| 8 | 19Del(0.56%), T790M(0.30%) | 19 Leu747_Pro753Del(0.36%), 20 T790M(0.63%), EGFR 23 Ser921Arg 44.50% | EGFR 23 Ser921Arg 77.05%, 19 Leu747_Pro753Del(53.68%), TP53 4 Cys124Ter (41.35%), 20 T790M (18.35%) | Invalid |
| 9 | L858R(1.76%) | 21 L858R(1.78%), TP53 6 Tyr220Cys(1.53%) | 21 L858R (29.38%), TP53 6 Tyr220Cys 33.33%, MET Amp 10.55 | L858R |
| 10 | L858R(0.07%) | 21 L858R(0.29%) | TP53 9 Leu308GlnfsTer27(28.07%), 21 L858R(59.28%), EGFR Amp（4.12）, MET Amp(7.81) | L858R |
| 11 | 19Del(11.1%), T790M(10.37%) | 19 Leu747_Pro753Del(47.89%), 20 T790M(16.7%), EGFR Amp（4.58） | 19 Leu747_Pro753Del(52.31%), ERBB2 Amp 10.60 | 19Del |
| 12 | 19Del(0.33%), L858R(0.16%), T790M(0.10%) | 19 Glu746_Ala750Del(0.72%), 21 L858R(0.44%), 20 T790M(0.31%) | 19 Glu746_Ala750Del(6.55%), TP53 c.376-1G>C(86.22%) | 19Del |
| 13 | 19Del(29.1%) | 19 Glu746_Ala750Del(62.42%), TP53 c.376-1G>C(72.72%) | 19 Glu746_Ala750Del(85.35%), TP53 c.376-1G>C(86.22%) | 19Del |
| 14 | Invalid | Invalid | MET 18 c.3632_3632+3del 8.05%, ALK EML4-ALK 2.33%, MET 15 Gln1017Ter 11.45% | Invalid |
| 15 | T790M（0.45%） | Invalid | Invalid | Invalid |
| 16 | Invalid | TP53 7 p.Arg249Ser(0.33%) | KRAS 2 Gly12Asp 48.26% TP53 5 Pro177His 41.21% | No mutation |
| 17 | 19Del(0.45%), T790M（0.39%） | 19 Glu746_Ala750Del 1.86%, 20 T790M 2.28%, TP53 8 p.Arg273Cys 0.58%, ALK 1 Arg133Cys 1.92%, PIK3CA 4 Cys255Arg 1.69% | 19 Glu746_Ala750Del 68.25%, 20 T790M 52.22%, TP53 7 Met243Ter 34.19%, EGFR Amp 5.57 | 19Del, T790M |
| 18 | 19Del 1.28%, T790M 0.87% | 19 Glu746_Ala750Del(2.02%), 20 T790M (0.74%), TP53 8 p.Cys277Phe (1.31%) | 19 Glu746_Ala750Del 68.25%, 20 T790M 52.22%, TP53 8 Cys277Phe 70.24%, PIK3CA Amp 4.18 | Invalid |
| 19 | Exon21-858 0.75% | 21 L858R 95.14%, TP53 c.376-1G>A 43.08%, TP53 7 Arg249Ser 0.12%, EGFR Amp 32.92 | 21 L858R 93.71%, EGFR Amp 16, TP53 c.376-1G>A 33.23% | L858R |
| 20 | 19Del 0.12% | 19 Glu746_Ala750Del 0.16% | 21 L858R 93.71%, MET Amp 5.87, TP53 6 His193Leu 31.18% | Invalid |
| 21 | T790M 0.3% | 21 L858R 0.23%, FGFR1 15 p.Asn690= 47.47% | 21 L858R 27.19%, TP53 10 Arg342Ter 24.22%, TP53 5 Lys132Thr 17.49%, FGFR1 15 Asn690= 59.82% | L858R |
| 22 | No EGFR mutation | EGFR 20 Asn771_Pro772insThr 1.22%, ALK 10 Arg606= 45.14% | EGFR 20 Asn771_Pro772insThr 26.74% , TP53 7 Cys242Phe 11.49%, ALK 10 Arg606= 41.87% | No EGFR mutation |
| 23 | Exon18-719 0.54 % | EGFR 18 p.Gly719Ala 5.89%, TP53 4 p.Ser95Tyr 2.27%, TP53 4 Ser96PhefsTer53 2.13%, ERBB2 Amp5.50 | 18 Gly719Ala 10.12%, TP53 4 p.Ser96PhefsTer53 6.68%, PIK3CA 2 p.Glu80Gly 4.96%, TP53 4 Ser95Tyr 8.00%, ERBB2 Amp 13.36 | 18 G719X |
| 24 | EGFR 21-858 1.02% | EGFR 21 p.Leu858Arg 1.21%, EGFR 25 p.Tyr998Cys 1.71%, TP53 3 Pro27LeufsTer17 1.30% | EGFR 21 p.Leu858Arg 10.97%, TP53 3 p.Pro27LeufsTer17 9.92%, EGFR 25 p.Tyr998Cys 12.34% | Invalid |
| 25 | No EGFR mutation | EGFR 20 c.2369C>T p.Thr790Met 0.21% | EGFR 19 p.Glu746_Ala750del 22.36%, EGFR 20 c.2369C>T p.Thr790Met 12.18% | Invalid |
| 26 | No EGFR mutation | MET c.2583+6A>G 52.49% | Invalid | Invalid |
| 27 | EGFR Exon21-858 0.08% | EGFR 20 p.Met766CysfsTer130 0.18%, FGFR1 5 p.Pro181Ser 52.47% | EGFR 21 p.Leu858Arg 29.36%, EGFR 20 p.Thr790Met 8.64%, EGFR 3 p.Arg108Lys 32.75%, FGFR1 5 p.Pro181Ser 46.92% | Invalid |
| 28 | EGFR Exon21-858 2.87%, EGFR Exon20-790 0.78% | EGFR 21 p.Leu858Arg 4.59%, EGFR 20 p.Arg776His 2.14%, EGFR 20 p.Thr790Met 1.18% | EGFR 21 c.2573T>G p.Leu858Arg 5.65%, EGFR 20 c.2369C>T p.Thr790Met 1.45% | No EGFR mutation |
| 29 | EGFR Exon19-del 1.03% | No mutation | EGFR 19 p.Glu746_Ser752delinsVal 16.07%, MET 16 p.His1094Leu 3.51% | 19del |
| 30 | EGFR Exon21-858 0.62%, EGFR Exon20-790 0.05% | EGFR 21 p.Leu858Arg 0.81%, EGFR 27 p.Leu1055= 50.52%, ALK 4 p.Ser341Gly 49.78% | EGFR 21 p.Leu858Arg 13.86%, EGFR 20 p.Thr790Met 4.09%, TP53 8 p.Asp281Gly 35.86%, ALK 4 p.Ser341Gly 56.04%, EGFR 27 p.Leu1055= 76.20%, EGFR Amp 6.17 | 21 L858R, 20 T790M |
| 31 | EGFR Exon19-del 2.8% | TP53 7 c.782_782+ 1delinsTT 1.58%, ALK 9 p.Trp593Leu 1.30% EGFR 6 p.Cys236Phe 1.33% | TP53 7 c.782_782+1delinsTT 50.93%， ALK 9 p.Trp593Leu 41.71%， EGFR 6 p.Cys236Phe 23.02% | Invalid |
| 32 | No EGFR mutation | No mutation | TP53 6 p.Arg213Ter 31.33% | No EGFR mutation |
| 33 | EGFR Exon21-858 1.35%, EGFR Exon20-790 0.07% | EGFR 21 p.Leu825Arg 0.46%, EGFR 20 p.Thr790Met 1.20%, TP53 7 p.Arg249Ser 0.11% | EGFR 21 p.Leu858Arg 60.12%， EGFR 20 p.Thr790Met 53.00%， TP53 c.559+1G>A 42.23% | 21 L858R， 20 T790M |
| 34 | EGFR Exon19-del 0.39%, EGFR Exon20-790 0.14% | EGFR 19 p.Glu746_Ala750del 0.53%, PIK3CA 10 p.Glu545Lys 0.40% | EGFR 19 p.Glu746_Ala750del 36.61%, PIK3CA 10 p.Glu545Lys 27.40%, TP53 8 p.Glu294Ter 77.40% | 19del |
| 35 | No EGFR mutation | EGFR 21 p.Leu861Gln 9.88% | TP53 4 p.Val73TrpfsTer50 21.79%, EGFR 21 p.Leu861Gln 81.30%, EGFR Amp 5.66 | No EGFR mutation |
| 36 | EGFR Exon19-del 0.36%, EGFR Exon21-858 2.48% | EGFR 19 p.Glu746_Ala750del 0.12%, EGFR 21 p.Leu858Arg 2.75%, TP53 c.560-2A>G 7.21%, MET 2 p.Ile284Val 61.69% | EGFR 21 p.Leu858Arg 8.81%, MET 2 p.Ile284Val 45.50%, TP53 c.560-2A>G 12.20%, ERBB2 Amp 8.51 | Invalid |
| 37 | EGFR Exon21-858 1.11%, EGFR Exon20-790 1% | EGFR 21 p.Leu858Arg 1.66%, EGFR 20 p.Thr790Met 2.36% | EGFR 21 p.Leu858Arg 22.33% | 21 L858R |
| 38 | No EGFR mutation | Invalid | EGFR 19 p.Glu746_Ala750del 79.88%, EGFR Amp 6.18 , MET Amp 4.55, TP53 5 p.Arg175His 41.71%, ALK c.3067+5G>A 47.14% | 19del |
| 39 | EGFR Exon19-del 5.2% | EGFR 19 p.Glu746_Ala750del 6.87%, TP53 5 p.Cys135Tyr 10.44% | EGFR 19 p.Glu746_Ala750del 23.68%, TP53 5 p.Cys135Tyr 42.32%, MET Amp 6.25 | 19del |
| 40 | No EGFR mutation | TP53 6 p.Tyr220Cys 4.43% | No mutation | No EGFR mutation |
| 41 | No EGFR mutation | No mutation | EGFR 21p.Leu858Arg 38.28%, EGFR 20 p.Thr790Met 5.16%, EGFR 2 p.Leu62Arg 38.68% | 21 L858R, 20 T790M |
| 42 | No EGFR mutation | No mutation | No mutation | No EGFR mutation |
| 43 | Invalid | Invalid | EGFR 21 p.Leu858Arg 49.31%, EGFR 20 p.Thr790Met 26.19%,ERBB2 amp 5.87 | Invalid |
| 44 | Invalid | Invalid | No mutation | No EGFR mutation |
| 45 | EGFR Exon21-8581.3% | KRAS 2 .Gly12Val 1.49% | EGFR 19 p.Glu746_Ala750del 18.73%, EGFR 20 Thr790Met 1.01%, TP53 7 p.Gly244Asp 16.40% | 19del, 20 T790M |
| 46 | EGFR Exon21-858 6.75%, EGFR Exon20-790 1.43% | EGFR 21 p.Leu858Arg 17.91%, EGFR 20 p.Thr790Met 8.70%,TP53 8 p.Glu286Lys 11.75%, TP53 7 p.Arg248Trp 0.73%, MET 7 p.Asn642Ser 21.92% | EGFR 21 p.Leu858Arg 31.57%, MET Amp 4.17, TP53 7 p.Arg248Trp 10.08% | 21 L858R |
| 47 | No EGFR mutation | No mutation | EGFR 21 p.Leu861Gln 22.05%, ERBB2 Amp 19.37, TP53 7 p.Ser241Cys 36.59%, ALK 4 p.Met378Ile 21.76% | No EGFR mutation |
| 48 | EGFR Exon21-858 1.44%, EGFR Exon20-790 0.66% | EGFR 21 p.Leu858Arg 3.74%, EGFR 20 p.Thr790Met 1.13%, PIK3CA 10 p.Glu545Lys 6.24%, TP53 8 p.Gly266Val 3.57% | EGFR 21 p.Leu858Arg 50.16%, EGFR 20 p.Thr790Met 15.94%, PIK3CA 10 p.Glu545Lys 55.58%, TP53 8 p.Gly266Val 61.19% | 21 L858R, 20 T790M |
| 49 | No EGFR mutation | EGFR 19 p.Glu746_Ala750del 0.15% | EGFR 19 p.Glu746_Ala750del 3.73% | 19del |
| 50 | EGFR Exon21-858 0.54% | EGFR 21 p.Leu858Arg 0.71%, PIK3CA 5 p.Asn345Lys 0.15%, TP53 7 p.Arg249Ser 0.72% | EGFR 21 p.Leu858Arg 13.60%, PIK3CA 10 p.Glu545Lys 11.73%, MET c.2584-7C>T 11.76% | 21 L858R |
