# Supplementary material for: Advanced NSCLC Patients With EGFR T790M Harboring TP53 R273C or KRAS G12V Cannot Benefit From Osimertinib Based on a Clinical Multicentre Study by Tissue and Liquid Biopsy
Source: Front Oncol. 2021 Feb 24;11:621992. doi: 10.3389/fonc.2021.621992 (PMC7943858; doi:10.3389/fonc.2021.621992)
Supplement: Supplementary file 2 [file Table_2.docx]

Supplementary Table 2. Summary of PFS to first-generation EGFR-TKIs in each patient

| Patient ID | PFS to PFS to first-generation EGFR-TKIs (month) |
| --- | --- |
| 1 | 19 |
| 2 | 8 |
| 3 | 30 |
| 4 | 12 |
| 5 | 3 |
| 6 | 12 |
| 7 | 13 |
| 8 | 14 |
| 9 | 15 |
| 10 | 6 |
| 11 | 13 |
| 12 | 31 |
| 13 | 13 |
| 14 | 10 |
| 15 | 4 |
| 16 | 22 |
| 17 | 35 |
| 18 | 14 |
| 19 | 2 |
| 20 | 6 |
| 21 | 9 |
| 22 | 15 |
| 23 | 6 |
| 24 | 21 |
| 25 | 52 |
| 26 | 63 |
| 27 | 16 |
| 28 | 8 |
| 29 | 31 |
| 30 | 12 |
| 31 | 2 |
| 32 | 16 |
| 33 | 19 |
| 34 | Undetermined |
| 35 | 6 |
| 36 | 26 |
| 37 | 27 |
| 38 | 9 |
| 39 | Undetermined |
| 40 | 19 |
| 41 | 7 |
| 42 | 6 |
| 43 | 17 |
| 44 | 3 |
| 45 | 5 |
| 46 | 16 |
| 47 | 16 |
| 48 | 19 |
| 49 | 9 |
| 50 | 19 |

PFS: progression-free survival
